# Enhancing musical creativity through AI: a chain-mediation model of self-efficacy and emotional intelligence

**DOI:** 10.3389/fpsyg.2025.1665609

**Published:** 2025-11-05

**Authors:** Tingzheng Wang, Junhan Zhang

**Affiliations:** ^1^School of Art, University of Bristol, Bristol, United Kingdom; ^2^Department of Music, Sejong University, Seoul, Republic of Korea

**Keywords:** musical creativity, artificial intelligence (AI), music composition, self-efficacy, emotional intelligence

## Abstract

**Introduction:**

The rapid advancement of AI technology is fundamentally transforming traditional music creation and influencing music education. However, whether and how the use of AI genuinely enhances musical creativity remains a subject for further exploration. This study aims to investigate the impact of AI usage on the musical creativity of music majors.

**Methods:**

Grounded in the “zone of proximal development” and social cognitive theory, this study constructed a theoretical model. The model introduced “musical self-efficacy” and “musical emotional intelligence” as mediating variables to explore the role of individual psychological factors. A quantitative approach was employed, utilizing questionnaire surveys and structural equation modeling (SEM) to analyze the relationships.

**Results:**

The findings revealed that: (1) The use of AI had a significant positive impact on enhancing musical creativity. (2) Musical emotional intelligence mediated the relationship between AI use and creativity, suggesting that individuals with higher emotional intelligence could better leverage AI. (3) Musical self-efficacy also served as a mediator, indicating that one’s belief in their own abilities influenced the effectiveness of AI tool use. (4) Music self-efficacy and music emotional intelligence acted as sequential mediators, highlighting the important moderating role of psychological factors between technology and creativity.

**Discussion:**

This study deepens the understanding of the relationship between AI technology and musical creativity. It provides practical guidance for higher music education, emphasizing the need to cultivate students’ self-efficacy and emotional intelligence in AI-assisted creative environments to achieve a synergistic development of technology and creativity.

## 1 Introduction

As a core feature of human cognitive abilities, musical creativity has always been a key topic in psychology and art research. Since Guilford defined creativity as “the ability to generate novel and applicable solutions to problems,” the concept has expanded from its initial psychometric dimension to interdisciplinary research fields (Su et al., 2018). Existing research indicates that musical creativity not only drives cultural innovation in the arts, but is also a driving force behind the process of contemporary social diversity (Qureshi, 2023). In the field of music composition, this creative practice exhibits a unique dual nature: it requires adherence to rigorous musical theory and formal aesthetics, while also demanding the breaking of established paradigms to achieve innovative expressions of emotional coding (Webster, 2002). This tension makes musical creativity an important part of exploring the essence of human creativity. At the same time, however, this dualistic situation raises a fundamental question: how can a creative balance be achieved between technical proficiency and emotional freedom?

The revolutionary breakthroughs in artificial intelligence technology have provided a new path for reconciling the above contradictions. AI music tools such as MusicVAE and Transformer music models have achieved systematic creative assistance ranging from melody generation and harmony configuration to emotional style transfer, systematically lowering the technical threshold for music creation (Roberts et al., 2018; Shih et al., 2022). Technical literature shows that current AI music systems can generate content of professional creative quality by capturing semantic features of music (Wu and Yang, 2023). The development of this technology has greatly expanded the boundaries of music creation and fundamentally challenged the traditional notion that “musical creativity is unique to humans.” It is worth noting that college students, with their dual advantages in digital technology acceptance and artistic innovation, have become a key research subject in exploring human-machine collaborative creation paradigms.

However, behind the controversy surrounding technological substitution lies a more revolutionary proposition: how can AI drive musical creativity to new heights by reconstructing the psychological cognitive mechanisms of creators? Based on social cognitive theory (Bandura, 1986), the effect of AI on enhancing musical creativity is not simply a matter of replacing tools, but rather a cognitive upgrade achieved by influencing individual psychological pathways. Specifically, this influence manifests itself through two mechanisms. First, musical self-efficacy acts as a psychological mediator of technological empowerment. AI tools significantly reduce the technical complexity of creation through functions such as real-time feedback (e.g., harmony error correction) and task decomposition (e.g., track generation) (Huang and Yang, 2020). This creative approach makes it easier for creators to accumulate successful experiences, thereby enhancing their confidence in their own musical abilities (Beghetto, 2019). For example, when creators use Amper Music’s style matching algorithm to complete cross-genre compositions, their sense of self-efficacy may be enhanced by the perception of “breaking through skill boundaries.” (Rezwana and Maher, 2023) This psychological reconstruction will directly influence the willingness to take creative risks, prompting creators to try more innovative forms of expression. Furthermore, AI technology indirectly influences musical creativity by reshaping creators’ cognitive abilities and emotional regulation patterns. Secondly, musical emotional intelligence acts as an emotional hub for human-machine collaboration. The emotional computing capabilities of AI tools provide college students with an explicit learning interface for “musical emotional grammar” (Livingstone et al., 2010). Furthermore, musical emotional intelligence functions as an affective nexus in human-computer collaboration. The affective computing capabilities of AI tools offer an explicit learning interface for “musical emotion grammar,” enabling creators to systematically analyze emotional encoding patterns – such as the association between minor scales and melancholic expressions (Vuoskoski et al., 2011). Through this analytical process, creators enhance their efficiency in translating emotional concepts into musical symbols. Notably, individuals with heightened self-efficacy demonstrate greater propensity to explore AI’s emotion regulation functionalities, and their successful exploratory experiences subsequently reinforce their emotional intelligence, establishing a mutually reinforcing cognitive cycle (Zimmerman, 2000).

Based on this, this study focuses on college students and constructs a theoretical model with music self-efficacy and emotional intelligence as chain mediators to explore the psychological pathway of AI use in music creation on music creativity. By revealing the cognitive upgrade mechanism in human-computer collaborative creation, this study not only fills the gap in psychological research on the application of AI music tools but also provides empirical evidence for innovative practices in college music education.

## 2 Literature review

### 2.1 The use of AI in music creation

Artificial intelligence (AI) is a technology that uses machines to simulate human intelligence to solve complex problems. The invention of digital computers, which made complex calculations efficient and accurate, led humans to begin exploring how to use computers for reasoning (Nehra, 2015). The basic concept of AI can be traced back to Turing’s article published in Mind magazine in 1950, which proposed the Turing test and sparked the debate “Can machines think?” (Turing, 1980). Since then, AI has gradually penetrated various fields. In music, the emergence of generative AI tools has had a huge impact on various sub-disciplines of music.

The core technology of AI in music creation is manifested in algorithmic composition systems. Liu and Ting (2016) systematically reviewed the application of evolutionary algorithms and neural networks in melody generation, rhythm design, and polyphonic arrangement, pointing out that these technologies can simulate compositional thinking through data-driven methods to generate complex musical structures. Recent technological developments have tended to combine multiple types of information (such as sound, images, and text) to assist AI in generating music. Deruty et al. (2022) verified the advantages of AI tools using audio signals as input in composition, arrangement, and mixing in the context of popular music production. These tools can directly process raw audio, providing composers with instant feedback, lowering technical barriers, and improving creative efficiency. In addition, Bretan and Weinberg (2016) interactive AI composition system, which dynamically generates accompaniment by analyzing the performer’s intentions in real time, further expands the boundaries of improvisation. AI is not intended to replace human creators, but rather to expand creative possibilities through distributed intelligence. Based on the theory of musical creativity (novelty, surprise, and value), Gioti (2020) compared the differences between humans and AI in music generation: humans are good at semantic expression based on cultural context, while AI can generate novel combinations beyond experience through massive data mining. Building on this, Zulić (2019) further pointed out through examples from the field of education that AI can serve as a “creative catalyst” to help novice composers break through skill limitations. For example, AI-assisted tools can generate multiple variations based on simple user input, or reveal potential harmonic paths through a visual interface, thereby accelerating learning and inspiring creativity. Such practices confirm that humans and AI can achieve creative value through complementary collaboration. Based on existing research, this paper will conduct empirical research to analyze how the use of AI in music creation affects individual musical creativity.

### 2.2 Musical self-efficacy

Self-efficacy refers to an individual’s assessment of their ability to successfully complete a task in a specific situation (Schwarzer and Luszczynska, 2008; Waddington, 2023). Bandura defined it as “an individual’s judgment of their ability to organize and execute actions to achieve predetermined performance goals.” (Bandura, 1986). This concept focuses on individuals’ subjective beliefs about their own abilities, rather than actual abilities or performance. Based on this, this paper focuses on musical self-efficacy in terms of individuals’ beliefs about their abilities in musical activities such as singing and playing an instrument. Bandura’s self-efficacy theory proposes four main sources: personal direct experience (successful experiences), vicarious experience, persuasion by others (social persuasion), and physiological and emotional states (Bandura, 2002). This framework provides a theoretical basis for understanding the personal and external factors that influence self-efficacy in the music learning process. In conjunction with the field of music, Zarza-Alzugaray et al. (2020) discussed the factors influencing music students’ performance self-efficacy, emphasizing the importance of both personal and environmental factors in achieving success. Research indicates that self-efficacy in music is closely related to performance outcomes and highly correlated with motivation levels. Studies suggest that personal motivation, emotional regulation, and learning environment are key factors influencing self-efficacy during the development of a music career. Similarly, Ritchie and Williamon’s (2012) study explored the relationship between self-efficacy and music performance quality. The study analyzed the relationship between self-efficacy, practice time, and self-regulated learning and assessment among university music majors and their performance quality. The results indicate that self-efficacy plays an important role in predicting students’ self-assessment of performance quality. The above researchers explored the relationship between self-efficacy and music performance from different dimensions. In addition, Hewitt’s research focuses on the dimension of successful experiences. The study explores how middle school band students tend to underestimate their performance as their performance skills improve (Hewitt, 2015). McPherson and McCormick’s (2006) research focused on the impact of self-efficacy on the performance abilities of young musicians, particularly its predictive role in graded music exams. Through structural equation modeling analysis, although there were differences in the results of the two types of music performance exams, self-efficacy remained the most important predictor of performance. The findings emphasize the critical role of self-efficacy in music performance and discuss the implications of this finding in the final section of the article. Jiang’s research explores the impact of music learning on students’ academic performance and mental health, and analyzes the mediating role of self-efficacy and self-esteem in this process (Jiang, 2024). The results indicate that music education can significantly improve students’ mental health, and this improvement further promotes their academic performance. In addition, the study also found that self-efficacy and self-esteem play an important mediating role in the relationship between mental health and music education.

### 2.3 Musical emotional intelligence

Emotional intelligence is considered to be the ability to accurately assess and express one’s own emotions and those of others, as well as the ability to effectively regulate one’s own emotions and those of others, and to use emotions to motivate, plan, and achieve personal goals (Neubauer and Freudenthaler, 2005). Emotional intelligence (EI) includes four key abilities: the ability to assess and express one’s own emotions, the ability to recognize and assess the emotions of others, the ability to manage one’s own emotions, and the ability to use emotions. These abilities help individuals understand and regulate their own emotions and those of others, promoting positive behavior and better personal performance (Law et al., 2004). As an important branch of emotional intelligence, musical emotional intelligence focuses on accurately assessing the emotional states of individuals and teams during musical activities, regulating and optimizing musical performance through immediate feedback, and promoting emotional communication and collaboration during the process of musical creation and performance. American psychologist Howard Gardner proposed the theory of multiple intelligences, which identifies eight distinct and relatively independent forms of intelligence. Musical intelligence is one of these, representing an individual’s ability to compose and appreciate musical elements such as rhythm, beat, and pitch. When applied to an individual, it manifests as skills in singing, playing musical instruments, and composing music (Gardner, 2008). Otchere (2014) investigated the relationship between music preference (MP) and emotional intelligence (EI). Through a mixed research design involving 100 undergraduate students, the study found that music type is significantly related to emotional intelligence. Fast-paced and traditional music types are positively correlated with emotional intelligence, while intense and rebellious music types are negatively correlated. In addition, movie soundtracks and theme songs are positively correlated with emotional intelligence, while rock music is negatively correlated. Similar research by Resnicow et al. (2004) explored the relationship between emotional intelligence (EI) and emotional recognition ability in musical performance. In the study, 24 undergraduates completed the Mayer-Salovey-Caruso Emotional Intelligence Test (MSCEIT) and a music emotion recognition task, which required students to identify emotions conveyed in classical piano performances. The results showed a significant positive correlation between emotional intelligence and emotion recognition ability in the music task, indicating that emotion recognition ability in music performance is associated with certain aspects of everyday emotional intelligence. Jia and Ayob (2025) explored the importance of emotional intelligence (EI) in musical performance and personal growth. Research has highlighted the importance of integrating emotional intelligence into music education. The core dimensions of EI (such as self-awareness and empathy) play a key role in improving musical expression, technical precision, and emotional connection. By incorporating EI strategies into music education, educators can enhance students’ artistic and emotional abilities, thereby reducing performance anxiety, increasing resilience, and promoting deeper connections between musicians and audiences. Gleason studied the relationship between musical ability and emotional intelligence and proposed that musical ability plays a mediating role in the effect of musical training on emotional intelligence (Gleason, 2014). Based on the above literature review, this paper focuses on musical intelligence and emotion as the ability of individuals to accurately assess and regulate their own and others’ emotions in musical activities, while promoting emotional communication and cooperation through musical expression. It combines musical intelligence and emotional intelligence to help individuals enhance the emotional depth and expressiveness of their musical composition and performance through emotional recognition and regulation in the process of playing, composing, and appreciating music.

### 2.4 Musical creativity

International scholars began studying musical creativity in the 1950s, 1940s, and 1930s. Musical creativity is the concentrated manifestation of various forms of practice and occupies a central position in music education. Existing research on musical creativity mainly focuses on professional musicians and composers (Burnard, 2012). Ryan and Brown (2012) explored the development and measurement of musical creativity in their research. Musical creativity is considered a skill that students develop during their growth, and although it has significant value in education, its definition remains vague. Musical creativity encompasses several different dimensions, including musical expansiveness, musical flexibility, musical uniqueness, and musical syntax (Burnard, 2012). Its scalability enables it to span different forms of creation and expression, not only in composition and improvisation, but also in non-traditional musical activities such as education and performance, demonstrating its potential for application in a variety of contexts (Bakht and Barlow, 2009). Flexibility is manifested in the ability of individuals to quickly adjust to different styles of music and quickly recognize different styles of musical works (Alward, 2023).

The uniqueness of music refers to the ability to express a strong personal style during the creative process, and uniqueness is also one of the major characteristics of innovative musical works (Monelle, 1997). Musical syntax involves various elements of music composition, such as melody, harmony, rhythm, and form. These musical elements require composers to follow certain formal and structural rules while innovating within these rules to produce novel and meaningful musical expressions (Gundlach, 1935).

In Runco’s article “The standard definition of creativity,” he proposed two important factors in musical creativity: creativity and originality. Creativity is an indispensable element of musical creativity and is often regarded as a manifestation of novelty or uniqueness. Original ideas and works, however, may still be useless or even meaningless if they lack practicality and effectiveness. Originality alone is insufficient to constitute the core of musical creativity. Truly creative musical works must not only be original but also effective, capable of demonstrating useful, appropriate, or suitable qualities in practical application (Runco and Jaeger, 2012). For a multidisciplinary study of musical creativity, international scholars Deliège and Wiggins first proposed the concept of musical creativity in their 2006 book “Musical Creativity: Multidisciplinary Research in Theory and Practice.” The book emphasizes the common interests of composers, performers, scholars, and others in this field. Although some progress has been made through interdisciplinary collaboration, the nature and origin of musical creativity remain an unsolved mystery, especially in the field of psychology, where research on musical creativity has yet to develop a unified theory (Deliège and Wiggins, 2006). Musical creativity is not merely a sudden flash of inspiration, but rather the innovative combination of information through ordinary cognitive processes such as reasoning, representation, association, working memory, and self-reflection. Within this framework, music composition is viewed as a complex cognitive activity in which different cognitive functions interact to produce new, meaningful ideas or experiences (López-González and Limb, 2012). Pachet (2006) discussed the challenges of researching musical creativity, particularly how to objectively measure musical creativity. He believes that although musical creativity can be expressed in works, traditional research methods often ignore its subjective experience and simplify it into objective evaluations. Pachet argues that musical creativity should be viewed from a subjective perspective as a personal creative experience, especially in interaction with computer systems. He proposes that this form of interaction is significant for understanding musical creativity. This perspective differs from traditional composition or performance, emphasizing the interaction between technology and the creative subject.

## 3 Research hypothesis

### 3.1 The use of AI can have a positive impact on enhancing college students’ musical creativity

The application of artificial intelligence music tools is profoundly changing the landscape of music creation. AI creation platforms such as Suno and ChatMusic provide students with a low-threshold, highly interactive creative environment through their intelligent melody generation and harmony arrangement functions. Chen’s research reveals the key mechanisms through which this technological intervention fosters musical creativity: AI tools, through instant feedback systems and personalized creative guidance, not only reduce technical barriers but, more importantly, stimulate students’ creative thinking (Chen, 2025). When students use AI tools for creative work, the diverse music style templates and real-time editing features provided by the system can effectively expand the boundaries of their musical imagination and encourage them to develop unique musical expressions through repeated trial and error. Zhang’s research in choral education further corroborates this finding, with data showing that students who used AI-assisted composition over a long period of time scored significantly higher on music originality assessments (Zhang, 2025).

The Zone of Proximal Development (ZPD) is an educational psychology concept proposed by Vygotsky, referring to the gap between the tasks that students can currently complete independently and the tasks that they can complete with external help (such as teachers or tools) (Shabani et al., 2010). This theory emphasizes that students can achieve higher cognitive levels with appropriate support than when learning independently, especially when faced with complex learning tasks. The theory mainly emphasizes that individuals can complete more difficult tasks with the help of external factors, such as chord progressions, structural layout, and motivational development. Similarly, Al-Ghawanmeh et al. (2014) proposed an automatic melody accompaniment generation method for Arabic improvised singing (Mawwāl) using informatics. The study analyzed the Mawaweel web model to identify the pivot notes of maqam and used these notes to generate accompaniment tracks, employing techniques such as sequential connection and glissando. Through comparative analysis with live performances, it was found that the model can effectively simulate improvised accompaniment, providing theoretical support for its application in AI music composition. Motukeeva et al. (2024) found that the use of digital educational tools significantly improved students’ creative thinking, especially increasing the proportion of high musical creativity and decreasing the proportion of low musical creativity. This indicates that digital technology has a positive impact on the development of students’ musical creativity. Based on ZPD theory, digital educational tools (AI) provide students with appropriate external support, helping them achieve higher levels of creative thinking within their potential capabilities. Through this support, students are able to transcend their current cognitive levels with the help of teachers or tools, thereby promoting the development of their musical creativity and complex thinking. Based on this, this paper proposes the following hypotheses:

*H1*: The use of AI can have a positive impact on enhancing college students’ musical creativity.

### 3.2 Musical emotional intelligence mediates between AI use and musical creativity

Technological innovations in AI music tools are reshaping the development mechanisms of musical creativity through the intermediary pathway of emotional intelligence. With the widespread application of AI-driven creation systems, the emotional mechanisms underlying music creation have undergone a transformation. These tools make the connections between musical elements and emotional characteristics explicit, such as decomposing the emotion of “sadness” into specific combinations of intervals and rhythmic patterns, enabling creators to intuitively understand and manipulate the emotional grammar of music. This technology significantly enhances creators’ musical emotional intelligence—the ability to identify, express, and regulate emotions through the musical notation system. Vadlamudi and Curha’s research shows that groups using AI tools for creation scored significantly higher on the MEI scale, and that this increase was significantly positively correlated with the creativity scores of their works (Vadlamudi and Curha, 2019). It is worth noting that AI technology has gender-differentiated effects on emotional intelligence. Jia and Ayob (2025) found that female creators are better at absorbing and applying the rules of AI emotional visualization tools, which may be related to the emotional expression of individuals of different genders during their growth process. In summary, AI technology is transforming emotional intelligence from a “talent-dependent” ability into a “technology-enhanced” skill, effectively broadening the demographic base for musical creativity. Based on this, this paper proposes the following hypothesis:

*H2*: Music emotional intelligence mediates the relationship between AI use and music creativity.

### 3.3 Musical self-efficacy mediates the relationship between AI use and musical creativity

The integration of artificial intelligence music tools is reshaping the development pathways of musical creativity through the psychological mechanism of self-efficacy. Based on social cognitive theory, the reinforcing effect of AI technology on music self-efficacy (i.e., an individual’s belief in their ability to complete music creation tasks) constitutes the core mediating pathway through which AI influences music creativity. This mechanism manifests specifically as follows: when students use AI music tools for creation, the system provides immediate positive feedback and task decomposition support (such as track-based generation), which reduces technical complexity, helps students accumulate successful experiences, and thereby enhances their self-efficacy levels (Wang and Li, 2024). Merrick’s research revealed that this psychological reinforcement enhances self-efficacy, which is closely related to students’ self-regulatory behavior during the creative process (Merrick, 2006). Students with high self-efficacy exhibit more and more complex self-regulatory behaviors, while those with low self-efficacy use these behaviors less frequently. During the process of completing tasks, students’ self-regulatory abilities also gradually improve, indicating that musical self-efficacy plays a crucial role in enhancing musical creativity. This interactive relationship was further validated in Lemons’ study on creative behavior, which collected data from 242 college students regarding their creative activities and their perceptions of their own creative abilities through an open-ended survey. The results showed that creative behavior is indeed related to creative self-efficacy (Lemons, 2010).

Bandura’s social cognitive theory provides a solid theoretical framework for the mediating mechanism through which AI music tools influence music creativity via music self-efficacy (Luszczynska and Schwarzer, 2015). This theory emphasizes that individuals actively shape behavioral pathways through self-regulation systems in interactions with their environment, while self-efficacy plays a central role in behavioral selection, effort, and persistence. In this theory, self-efficacy, as a core cognitive variable, demonstrates a significant mediating effect in music creation scenarios involving AI technology. Wang and Li (2024) found in their study of Chinese music students that self-efficacy and AI technology readiness can explain up to 63% of the variance in academic performance. These results reveal the intertwined relationship between individuals’ intrinsic beliefs and their ability to adopt technology. They also suggest that when studying the impact of AI music tools on musical creativity, we must consider musical self-efficacy as a potential key mediating variable. When music students have greater confidence in their own creative and expressive abilities, they are more likely to actively try and effectively use AI tools for music creation, thereby demonstrating higher levels of actual creative output. Zarza-Alzugaray et al. (2020) further revealed the formation mechanism of musical self-efficacy from the perspectives of social support and emotional factors. They used structural equation modeling to demonstrate that social support from family, teachers, and peers enhances students’ self-efficacy by alleviating anxiety related to musical performance. The study also found gender differences in the self-efficacy construction pathway, indicating that individual differences should be incorporated into teaching considerations. Extending this finding to the context of AI music creation, it suggests that when individuals are exposed to AI tools in an environment with sufficient social support, psychological comfort, and emotional stability, they are more likely to develop a sense of control and confidence in the music creation process. Based on social cognitive theory, we believe that musical self-efficacy is not only a bridge between AI music tools and musical creativity, but also a psychological variable that plays a key moderating and mediating role in this mechanism. Musically self-efficacious learners are more likely to actively embrace emerging technologies and engage in experimental attempts and stylistic innovations with the help of AI tools. This process further reinforces their self-efficacy through the continuous acquisition of “mastery experiences”—i.e., feedback from actual creative successes—forming a positive feedback loop. Conversely, individuals with low self-efficacy may fail to fully leverage the potential of AI tools, even if they possess technical readiness, due to a lack of intrinsic motivation. Based on this, the following hypotheses are proposed:

*H3*: Music self-efficacy mediates the relationship between AI use and musical creativity.

### 3.4 The mediating role of musical self-efficacy and musical emotional intelligence in the influence of AI use on musical creativity

**Figure 1:**
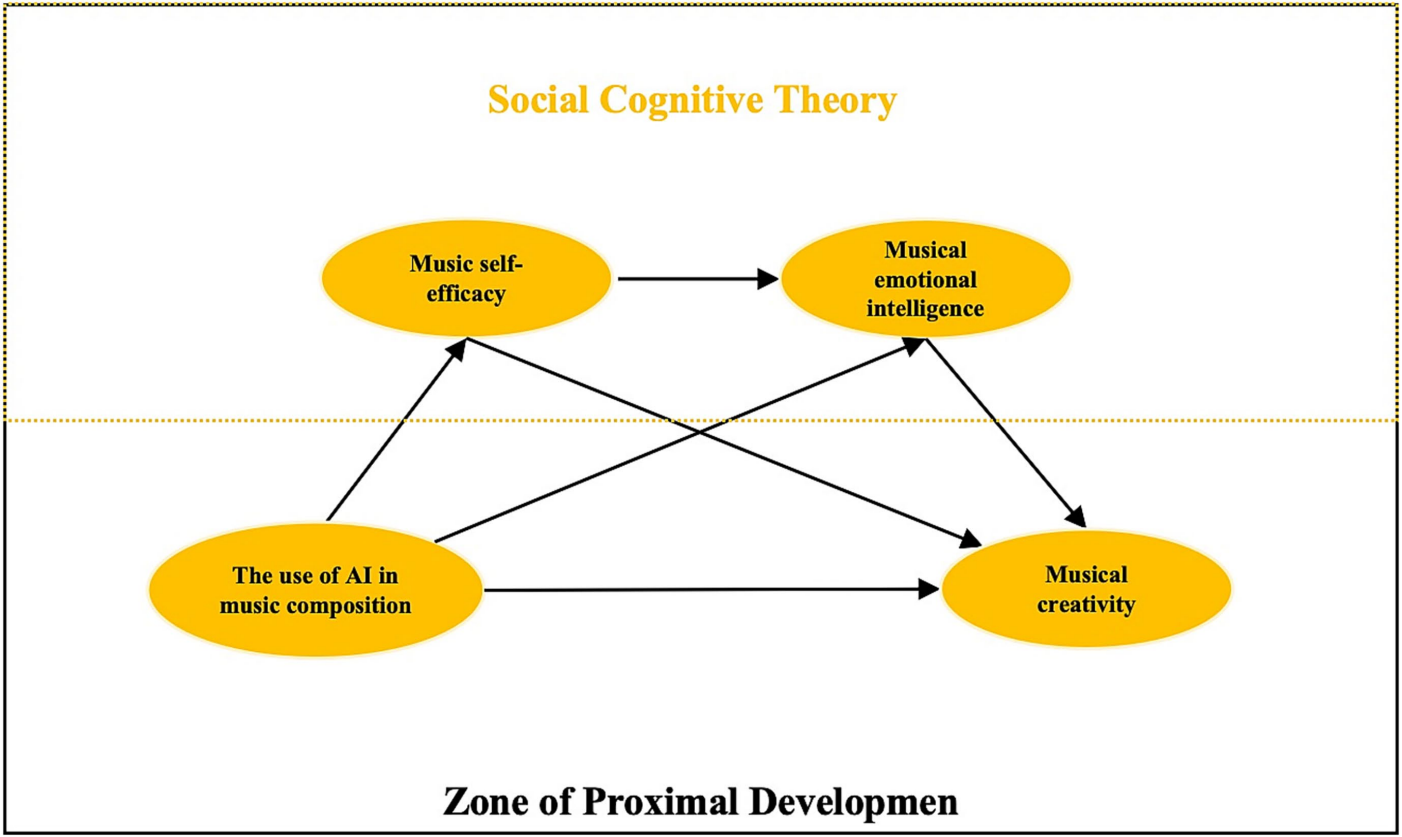
Proposed modeling diagram.

Based on the Zone of Proximal Development theory and social cognitive theory, this paper proposes the following chained mediation model, in which the use of AI positively influences music creativity through music self-efficacy and music emotional intelligence. First, as the usage of AI music tools increases, creators gain immediate feedback and successful experiences, leading to a significant improvement in their music self-efficacy—that is, their confidence in their music creation abilities. This enhanced self-efficacy not only directly promotes creative motivation and exploratory behavior but also further stimulates creators to actively utilize the emotional computing functions of AI tools, thereby improving their ability to identify, express, and regulate musical elements and emotions. Ultimately, this enhanced emotional intelligence enables creators to more accurately transform internal emotions into innovative musical expressions, thereby positively influencing musical creativity. Based on this, this paper proposes the following hypotheses (the proposed model diagram is shown in Figure 1):

*H4*: Music self-efficacy and musical emotional intelligence mediate the effect of AI use on musical creativity.

## 4 Research design

### 4.1 Recipient and questionnaire distribution

This study focuses on college students aged 18–22 and uses a stratified random sampling method to conduct offline surveys at six comprehensive universities (Tongji University, Shanghai University, Shaanxi Normal University, Xihua Normal University, Hunan University) and three colleges (Shaanxi Art Vocational College, Xianyang Vocational and Technical College, Shaanxi Youth Vocational College) in China. The study participants must be 18 years of age or older, have used AI music creation tools at least once in the past 3 months, and be students majoring in music-related fields (both theoretical and practical specializations are acceptable). Prior to the formal survey, the research team conducted a small-scale preliminary survey in early January 2025, distributing 30 initial questionnaires randomly at Shaanxi Normal University and recovering 26 completed questionnaires. Eight respondents were invited to participate in focus group discussions to address issues such as unclear or difficult-to-understand expressions in the questionnaire. Therefore, these 26 samples were excluded from the final statistical analysis. Finally, semantic clarification and adjustments were made to complex expressions in the questionnaire, such as “self-assessment of musical creativity” and “dependence on AI tools.”

The formal survey was conducted from January to May 2025. The research team visited nine universities and recruited participants on-site through campus exhibitions and other means. Questionnaires were distributed to music majors at different educational levels (vocational, undergraduate, and graduate), with an average completion time of 10–15 min. To strictly protect participants’ rights, the study was conducted entirely anonymously, and anonymity was ensured at every stage of data processing: First, during the informed consent process, participants were explicitly advised that they could use a pseudonym to sign, and the signature would only serve to indicate their understanding of the study content and voluntary participation. Second, all participants were informed that they retained the right to unconditionally withdraw from the survey at any time after signing the consent form. Finally, the collected paper questionnaires were securely stored and sealed by the corresponding author, and no other researchers had access to the original documents, thereby fully safeguarding participant privacy (Table 1).

**Table 1:** Basic information description and analysis.

Variable	Option	Frequency	Proportion (%)
Gender	Male	526	46.76
	Female	599	53.24
Age	18–19	332	29.51
	20–22	793	70.49
Educational background	Associate degree	346	30.76
	Bachelor’s degree	659	58.58
	Master’s degree	120	10.67
Hometown	Urban	449	39.91
	Rural	676	60.09
Living expenses (CNY)	1,000–1,500	443	39.38
	1,500–2000	300	26.67
	2000–3,000	257	22.84
	3,000 and above	125	11.11
Music major	Theoretical direction (composition, music history)	116	10.31
	Music education	229	20.36
	Instrumental performance (Western music, Chinese traditional music)	334	29.69
	Vocal performance (bel canto, ethnic, pop)	446	39.64

### 4.2 Variable measurement

The core variables in this study were measured using standardized scales that have been validated by the academic community. All items were quantified using a five-point Likert scale, with the following scale settings: 1 represents “strongly disagree,” 2 represents “somewhat disagree,” 3 represents “neutral,” 4 represents “somewhat agree,” and 5 represents “strongly agree.” All scales have undergone cross-cultural adaptation adjustments to ensure the alignment of measurement dimensions with the research context.

(1) The Use of AI in Music Composition: This study is based on the ChatGPT usage scale theoretical framework proposed by Nemt-Allah et al. (2024), and systematically revised it to address the specific characteristics of music composition. While retaining the original scale, it was adapted to the music composition context and developed into three core dimensions, including creative composition assistance, music production support, and dependence on and trust in AI output, resulting in a music composition-specific AI application assessment tool with a total of 15 items.

(2) Musical self-efficacy: This study adopts the three-dimensional measurement system of musical self-efficacy constructed by Ritchie and Williamon (2011), whose core dimensions cover ability confidence, goal persistence, and goal achievement and problem solving, systematically assessing self-efficacy in musical practice. On this basis, appropriate modifications are made in accordance with the research subjects of this paper, retaining 14 items as the measurement items for this paper.

(3) Musical Emotional Intelligence: This study draws on the emotional intelligence theoretical framework constructed by Law et al. (2004) and builds upon it to develop a four-dimensional assessment system for musical emotional intelligence. Convert the social context elements in the original model into musical practice scenarios to form a measurement system comprising 16 standardized items: self-awareness of emotions and artistic expression in musical contexts, decoding of others’ emotions and aesthetic empathy in musical interactions, dynamic regulation of emotions in musical activities, and conversion of emotional resources in musical creation.

(4) Musical Creativity: Musical creativity is the ability to transform novel and valuable ideas into reality. In the field of music, it manifests as a comprehensive set of skills encompassing composition, performance interpretation, and improvisation. Given the focus of this study on the field of music composition, a systematic literature review reveals that existing methods for measuring musical creativity are dominated by experimental approaches, while self-report questionnaire assessment tools are relatively underutilized in both methodological applications and empirical research. To address this research gap, this study integrates the core constructs of existing mature scales. Starting from Jiang ‘s Music Creativity Practice Ability Scale, it incorporates Doppelt ‘s Creative Thinking Scale and Kaufman ‘s Music Creativity Scale, and combines them with the research content of this paper to form a five-item condensed version of the Music Creativity Scale. The scale includes five items: originality, improvisation ability, expressiveness, collaboration ability, and creative strategies (Jiang et al., 2024; Doppelt, 2009; Kaufman, 2012).

The Cronbach’s Alpha coefficients were 0.914, 0.918, 0.846, 0.867, 0.903, 0.921, 0.895, 0.893, 0.887, 0.893, 0.924, and 0.937, all of which were greater than 0.7. This indicates that the scale is reliable and suitable for use. To ensure the scientific validity and applicability of the scale, the study invited five experts in the field of music with the title of professor and a doctoral degree to review the scale.

## 5 Model analysis

### 5.1 Common method bias

This study used Harman’s single-factor test to examine common method bias. The results showed that there were 11 factors with eigenvalues greater than 1, with a total explained variance of 74.30%, and the first principal factor explained 8.928% of the variance, which was less than the critical standard of 40%. Therefore, this study did not find any serious common method bias (as shown in Table 2).

**Table 2:** Total variance explained.

Component	Initial eigenvalues	Extraction sums of squared loadings	Rotation sums of squared loadings	Component	Initial eigenvalues
12.479	24.958	24.958	4.464	8.928	8.928
4.764	9.527	34.485	4.387	8.773	17.701
4.512	9.024	43.509	4.357	8.713	26.415
2.729	5.457	48.967	3.699	7.398	33.812
2.449	4.899	53.866	3.614	7.228	41.040
2.042	4.084	57.949	3.083	6.166	47.206
1.792	3.584	61.534	3.071	6.141	53.347
1.670	3.340	64.874	3.027	6.054	59.401
1.664	3.329	68.202	2.974	5.948	65.350
1.565	3.130	71.332	2.268	4.537	69.886
1.485	2.970	74.302	2.208	4.416	74.302

Extraction Method: Principal Component Analysis. Only the first 11 components with eigenvalues greater than 1 are displayed, cumulatively explaining 74.302% of the variance.

### 5.2 Exploratory factor analysis

Exploratory factor analysis was conducted using SPSS 23.0 to perform KMO and EArtlett’s sphericity tests on the questionnaire. The results are presented in Table 3. The KMO value was 0.938 > 0.7, and the EArtlett’s sphericity test was significant (Sig. < 0.001), indicating that the questionnaire data met the prerequisites for factor analysis. Therefore, further analysis was conducted. Principal component analysis was used for factor extraction, with eigenvalues greater than 1 as the criterion for selecting common factors. Orthogonal rotation with maximum variance was employed for factor rotation during factor analysis. The analysis results are shown in Table 2, with the total explanatory power reaching 74.302% > 50%, indicating that the selected 11 factors have good representativeness. As shown in Table 4, the factor loadings of all measurement items are greater than 0.5, and the cross-loadings are all less than 0.4. Each item falls into the corresponding factor, demonstrating good structural validity.

**Table 3:** KMO analysis table.

KMO and Bartlett’s Test		
Kaiser–Meyer–Olkin measure of sampling adequacy		0.938
Bartlett’s test of sphericity	Approx. Chi-square	37154.842
	df	1,225
	Sig.	0.000

**Table 4:** Rotated component matrix.

Rotated component matrix^a^											
	Component										
	1	2	3	4	5	6	7	8	9	10	11
AIMC1	0.049	0.157	0.779	0.058	0.127	0.008	0.017	0.019	0.025	0.002	0.082
AIMC1	0.049	0.153	0.809	0.010	0.084	0.019	0.075	0.085	0.035	0.016	0.126
AIMC1	0.043	0.126	0.787	0.049	0.124	0.031	0.050	0.043	0.096	0.048	0.063
AIMC1	0.065	0.130	0.827	0.028	0.131	0.046	0.089	0.054	0.055	0.024	0.098
AIMC1	0.009	0.123	0.805	0.035	0.102	0.013	0.057	0.107	0.066	0.041	0.092
AIMC1	0.059	0.148	0.809	0.031	0.096	0.038	0.078	0.040	0.079	0.032	0.096
AIMC2	0.072	0.813	0.123	0.031	0.114	0.057	0.052	0.048	0.066	0.038	0.099
AIMC2	0.089	0.830	0.136	0.043	0.114	0.031	0.049	0.057	0.054	0.033	0.077
AIMC2	0.040	0.782	0.155	0.075	0.087	0.045	−0.006	0.023	0.034	0.037	0.110
AIMC2	0.046	0.818	0.145	−0.006	0.061	0.038	0.089	0.042	−0.006	0.026	0.085
AIMC2	0.050	0.821	0.107	0.054	0.092	0.028	−0.001	0.036	0.055	0.024	0.064
AIMC2	0.094	0.821	0.162	0.026	0.087	0.017	0.043	−0.007	0.027	0.019	0.110
AIMC3	0.005	0.169	0.185	0.075	0.122	0.054	0.014	0.023	0.065	0.038	0.819
AIMC3	0.057	0.184	0.173	0.030	0.111	0.041	0.078	0.059	0.041	0.015	0.816
AIMC3	0.026	0.190	0.189	0.068	0.155	0.061	0.081	0.039	0.077	0.048	0.814
MSE1	0.196	0.046	0.056	0.202	0.114	0.064	0.053	0.042	0.034	0.820	0.062
MSE1	0.164	0.072	0.048	0.186	0.144	0.064	0.062	0.043	0.036	0.832	0.010
MSE1	0.194	0.046	0.042	0.209	0.104	0.061	0.053	0.042	0.058	0.831	0.030
MSE2	0.192	0.020	0.031	0.797	0.076	0.036	0.037	0.019	0.047	0.130	0.045
MSE2	0.145	0.054	0.050	0.816	0.086	0.087	0.024	0.060	0.052	0.127	0.046
MSE2	0.173	0.023	0.064	0.829	0.082	0.040	0.051	0.050	0.029	0.108	0.032
MSE2	0.147	0.083	0.033	0.805	0.081	0.073	0.025	0.003	0.038	0.107	0.032
MSE2	0.164	0.032	0.024	0.818	0.119	−0.013	0.040	0.048	0.052	0.098	0.025
MSE3	0.812	0.049	0.086	0.120	0.124	0.015	0.038	0.019	0.033	0.091	0.016
MSE3	0.827	0.076	0.045	0.125	0.075	0.073	0.057	0.044	0.087	0.062	−0.003
MSE3	0.816	0.040	0.060	0.156	0.084	0.042	0.009	0.023	0.035	0.140	0.060
MSE3	0.801	0.087	0.004	0.125	0.103	0.009	0.053	0.055	0.061	0.119	−0.016
MSE3	0.824	0.088	0.038	0.161	0.110	0.085	−0.004	0.044	0.022	0.055	0.027
MSE3	0.811	0.055	0.042	0.170	0.131	0.017	0.047	0.048	0.100	0.093	0.026
MEI1	0.039	0.056	0.049	0.017	0.137	0.810	0.151	0.173	0.144	0.068	0.050
MEI1	0.076	0.045	0.033	0.072	0.182	0.822	0.114	0.131	0.115	0.046	0.028
MEI1	0.067	0.033	0.034	0.077	0.133	0.818	0.129	0.151	0.153	0.038	0.057
MEI1	0.045	0.075	0.021	0.061	0.123	0.808	0.152	0.158	0.166	0.047	0.032
MEI2	0.070	0.069	0.109	0.015	0.106	0.134	0.810	0.135	0.161	0.021	0.023
MEI2	0.003	0.040	0.094	0.047	0.128	0.117	0.826	0.159	0.162	0.038	0.046
MEI2	0.063	0.054	0.074	0.062	0.108	0.145	0.808	0.171	0.159	0.073	0.087
MEI2	0.052	0.054	0.080	0.057	0.088	0.148	0.805	0.143	0.170	0.044	0.030
MEI3	0.067	0.060	0.079	0.053	0.091	0.145	0.138	0.810	0.139	0.030	0.039
MEI3	0.029	0.028	0.080	0.023	0.131	0.162	0.171	0.812	0.141	0.052	0.013
MEI3	0.063	0.043	0.111	0.043	0.137	0.156	0.138	0.810	0.159	0.023	0.008
MEI3	0.058	0.056	0.067	0.060	0.123	0.142	0.153	0.793	0.134	0.028	0.068
MEI4	0.077	0.051	0.062	0.057	0.120	0.177	0.206	0.172	0.786	0.030	0.031
MEI4	0.090	0.058	0.120	0.054	0.146	0.156	0.171	0.136	0.796	0.037	0.059
MEI4	0.106	0.070	0.078	0.078	0.161	0.128	0.197	0.205	0.801	0.053	0.044
MEI4	0.075	0.056	0.105	0.049	0.148	0.152	0.129	0.112	0.794	0.026	0.070
MC1	0.145	0.129	0.197	0.098	0.767	0.121	0.114	0.109	0.109	0.114	0.113
MC1	0.188	0.131	0.157	0.121	0.774	0.159	0.072	0.110	0.139	0.097	0.086
MC1	0.123	0.146	0.157	0.122	0.794	0.138	0.109	0.134	0.146	0.080	0.091
MC1	0.163	0.156	0.135	0.132	0.766	0.130	0.131	0.129	0.128	0.079	0.115
MC1	0.151	0.127	0.180	0.096	0.776	0.162	0.108	0.120	0.144	0.094	0.096

Extraction method: principal component analysis.

Rotation method: varimax with Kaiser normalization.

^a^Rotation converged in 7 iterations.

### 5.3 Confirmatory factor analysis

This study includes six types of variables, comprising a total of 50 measurement items. After conducting confirmatory factor analysis using AMOS 26.0, the results are presented in Table 5. The standardized factor loadings for all measurement indicators of each variable are greater than 0.6, the composite reliability (CR) is greater than 0.7, and the average variance extracted (AVE) is greater than 0.5, indicating that all variables exhibit good convergent validity. This study employed the rigorous AVE method to assess discriminant validity. For each factor, the square root of the AVE must be greater than the correlation coefficient between each pair of variables, indicating that the factors possess discriminant validity (Fornell and Larcker, 1981). The square root of the AVE for each factor is greater than the standardized correlation coefficient outside the diagonal, so this study still has discriminant validity (as shown in Table 6, with the slanted triangle representing the correlation coefficient).

**Table 5:** Confirmatory factor analysis results.

Variable	RC2	CR	AVE
AIMC1 (1)	0.779	0.914	0.641
AIMC1 (2)	0.809		
AIMC1 (3)	0.787		
AIMC1 (4)	0.827		
AIMC1 (5)	0.805		
AIMC1 (6)	0.809		
AIMC2 (1)	0.813	0.919	0.654
AIMC2 (2)	0.83		
AIMC2 (3)	0.782		
AIMC2 (4)	0.818		
AIMC2 (5)	0.821		
AIMC2 (6)	0.821		
AIMC3 (1)	0.819	0.848	0.651
AIMC3 (2)	0.816		
AIMC3 (3)	0.814		
MSE1 (1)	0.82	0.868	0.688
MSE1 (2)	0.832		
MSE1 (3)	0.831		
MSE2 (1)	0.797	0.903	0.652
MSE2 (2)	0.816		
MSE2 (3)	0.829		
MSE2 (4)	0.805		
MSE2 (5)	0.818		
MSE3 (1)	0.812	0.922	0.663
MSE3 (2)	0.827		
MSE3 (3)	0.816		
MSE3 (4)	0.801		
MSE3 (5)	0.824		
MSE3 (6)	0.811		
MEI1 (1)	0.81	0.896	0.683
MEI1 (2)	0.822		
MEI1 (3)	0.818		
MEI1 (4)	0.808		
MEI2 (1)	0.81	0.895	0.682
MEI2 (2)	0.826		
MEI2 (3)	0.808		
MEI2 (4)	0.805		
MEI3 (1)	0.81	0.888	0.664
MEI3 (2)	0.812		
MEI3 (3)	0.81		
MEI3 (4)	0.793		
MEI4 (1)	0.786	0.894	0.68
MEI4 (2)	0.796		
MEI4 (3)	0.801		
MEI4 (4)	0.794		
MC (1)	0.767	0.924	0.71
MC (2)	0.774		
MC (3)	0.794		
MC (4)	0.766		
MC (5)	0.776		

The numbers outside the brackets represent dimensions, and the numbers inside the brackets represent question items.

**Table 6:** Distinguishing validity and correlation analysis.

Dimension	AIMC1	AIMC2	AIMC3	MSE1	MSE2	MSE3	MEI1	MEI2	MEI3	MEI4	MC
AIMC1	0.801										
AIMC2	0.393	0.808									
AIMC3	0.430	0.416	0.807								
MSE1	0.167	0.173	0.178	0.829							
MSE2	0.152	0.154	0.186	0.465	0.807						
MSE3	0.174	0.213	0.143	0.420	0.430	0.814					
MEI1	0.164	0.174	0.213	0.229	0.198	0.197	0.826				
MEI2	0.263	0.187	0.235	0.210	0.173	0.172	0.438	0.826			
MEI3	0.253	0.171	0.194	0.188	0.173	0.186	0.474	0.475	0.815		
MEI4	0.271	0.205	0.253	0.216	0.216	0.256	0.477	0.524	0.493	0.824	
MC	0.422	0.369	0.411	0.379	0.343	0.395	0.454	0.393	0.419	0.475	0.843

### 5.4 Structural equation modeling analysis

**Figure 2:**
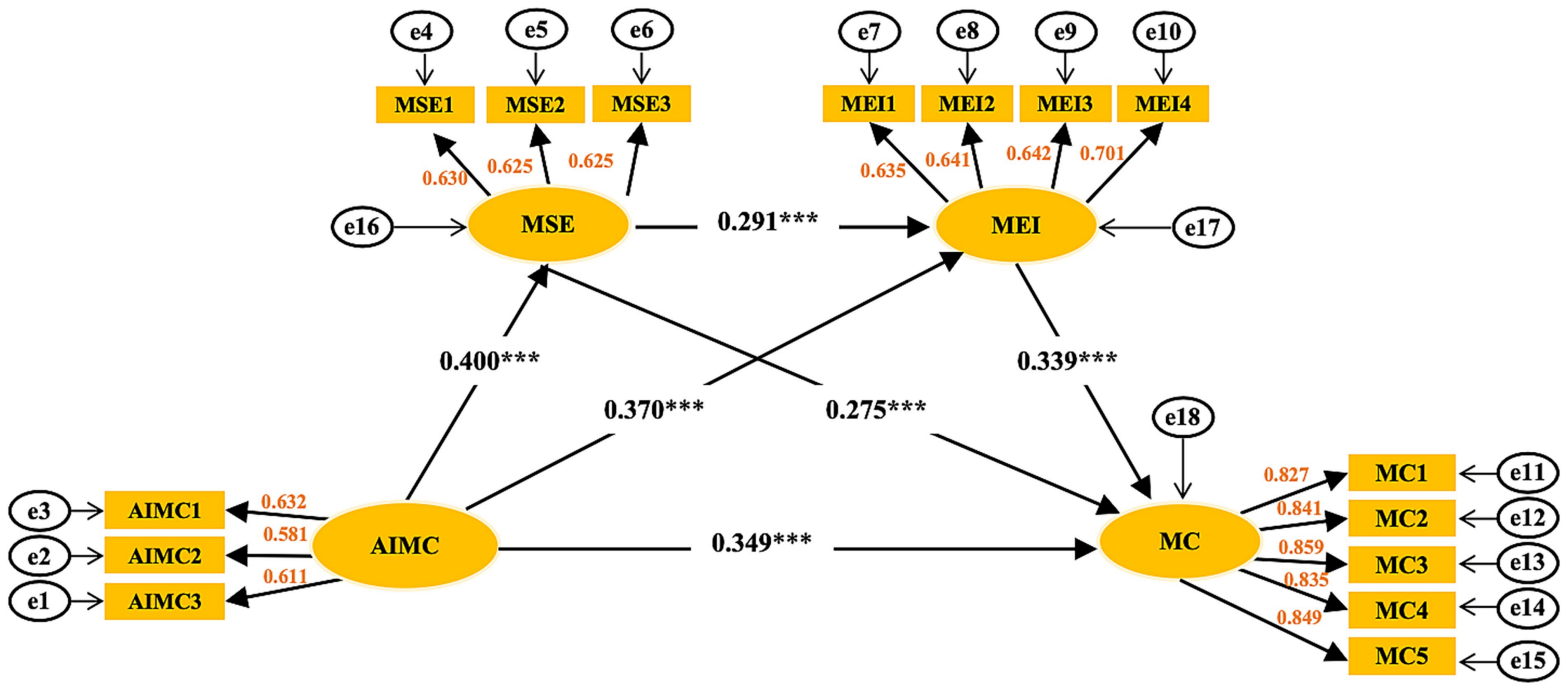
Structural equation analysis diagram. ***p < 0.001.

Using AMOS 23.0 for calculations and the maximum likelihood method for estimation, the results are shown in Figure 2. From the fit indices (Table 7), CMIN/DF is 1.548, which is below the standard of 3, and GFI, AGFI, NFI, TLI, IFI, and CFI all meet the standard of 0.9 or above. RMR is 0.029 < 0.08, and RMSEA is 0.033 < 0.08. All fit indices meet general research standards, so it can be concluded that the model has good fit.

**Table 7:** Model fitting index.

CMIN/DF	GFI	AGFI	RMR	RMSEA	NFI	IFI	CFI	TLI
1.548	0.958	0.952	0.016	0.010	0.967	0.997	0.997	0.996

The table (see Table 8) presents the unstandardized and standardized coefficients from the structural equation model (SEM) path analysis, interpreted as follows: (1) AIMC → MSE path: Unstandardized coefficient 0.428 (S. E. = 0.055, C. R. = 7.73, *p* = 0.001), standardized coefficient 0.4, indicating that AIMC has a significant positive effect on MSE an increase of 1 unit in AIMC leads to an increase of 0.428 units in MSE; (2) MSE → MEI path: Unstandardized coefficient 0.292 (S. E. = 0.049, C. R. = 5.945, *p* = 0.001), standardized coefficient 0.291, indicating that MSE has a significant but weaker effect on MEI than AIMC→MSE; (3) AIMC→MEI path: unstandardized coefficient 0.397 (S. E. = 0.056, C. R. = 7.133, *p* = 0.001), Standardized coefficient 0.37, indicating a direct effect of AIMC on MEI, which is slightly higher than MSE → MEI; (4) MEI → MC path: Unstandardized coefficient 0.465 (S. E. = 0.055, C. R. = 8.471, *p* = 0.001), Standardized coefficient 0.339, MEI has a significant effect on MC and is the strongest among all mediating paths; (5) AIMC → MC direct effect: Unstandardized coefficient 0.515 (S. E. = 0.064, C. R. = 8.078, *p* = 0.001), Standardized coefficient 0.349, indicating that the direct effect of AIMC on MC is greater than the indirect effect (MSE/MEI mediation); 6. MSE → MC path: Unstandardized coefficient 0.38 (S. E. = 0.054, C. R. = 7.052, *p* = 0.001), standardized coefficient 0.275, indicating that MSE has a partial mediating effect on MC independent of MEI. In summary, AIMC influences MC through the dual mediating effects of MSE and MEI, while retaining a significant direct effect. The model exhibits partial mediation.

**Table 8:** Path coefficient table.

Path coefficient table	Non-standardized coefficient	S. E.	C. R	*P*	Significance	Standardized coefficient
MSE ← AIMC	0.428	0.055	7.73	0.001	***	0.4
MEI ← MSE	0.292	0.049	5.945	0.001	***	0.291
MEI ← AIMC	0.397	0.056	7.133	0.001	***	0.37
MC ← MEI	0.465	0.055	8.471	0.001	***	0.339
MC ← AIMC	0.515	0.064	8.078	0.001	***	0.349
MC ← MSE	0.38	0.054	7.052	0.001	***	0.275

***p < 0.001.

### 5.5 Intermediate inspection

The mediation analysis in the research model confirmed all four hypotheses regarding the influence of AIMC on college students’ MC, with music self-efficacy (MSE) and music emotional intelligence (MEI) serving as mediating factors. The results of the sequential mediation effects are shown in Table 9.

**Table 9:** Chain mediation effect test.

Chain mediation effect test		Estimate	Lower	Upper	*P*
Direct effect	AIMC-MC	0.515	0.404	0.635	0.014
Indirect effect	AIMC-MSE-MC	0.162	0.122	0.212	0.007
	AIMC-MEI-MC	0.185	0.14	0.234	0.009
	AIMC-MSE-MEI-MC	0.058	0.038	0.084	0.006
Total effect	AIMC-MSE-MC	0.678	0.564	0.802	0.015
	AIMC-MEI-MC	0.7	0.575	0.822	0.015
	AIMC-MSE-MEI-MC	0.573	0.459	0.692	0.012

The direct effect of AIMC on MC (estimated value = 0.515, *p* = 0.014) is statistically significant and positive, confirming that AIMC directly promotes college students’ musical creativity, thus validating H1. The indirect effect of MSE (estimated value = 0.162, *p* = 0.007) is significant, indicating that MSE mediates the relationship between AIMC and MC. This supports the mediating role of musical self-efficacy in the relationship between AIMC and college students’ musical creativity. Therefore, H2 is valid. The indirect effect of MEI (estimate = 0.185, *p* = 0.009) indicates that MEI is a significant mediator. This finding suggests that AIMC positively influences college students’ musical creativity by affecting their musical emotional intelligence. Therefore, H3 is valid. The chained mediating effect of MSE and MEI (estimated value = 0.058, *p* = 0.006) is statistically significant. This result emphasizes a sustained mediating effect, where AIMC increases MSE, which in turn increases MEI, ultimately leading to an increase in MC. Therefore, H4 holds.

The total effect integrates the direct and mediating paths, further strengthening these findings and demonstrating that AIMC has a significant overall impact on MC through these mediating variables. Both the direct and mediating paths contribute to explaining the dynamic relationship proposed in the hypothesis, emphasizing the important role of musical self-efficacy and musical emotional intelligence in enhancing college students’ musical creativity under the influence of AIMC.

## 6 Discussion

### 6.1 Direct effect

The present study confirms that the use of AI exerts a significant direct facilitative effect on the enhancement of musical creativity. This finding should not be merely attributed to improvements in technical efficiency, but rather understood as AI functioning as a “cognitive collaborator” that restructures the process of creative cognition. Specifically, deep learning-based generative algorithms (e.g., MusicVAE, Transformer) expand the creator’s “conceptual space” by providing combinatorial possibilities that transcend the boundaries of traditional musical syntax. When creators interact in real-time with these algorithms, they are no longer merely executing preconceived musical ideas, but are continuously discovering new musical possibilities through dialog with the system.

Concurrently, by transforming technical challenges such as harmony and musical form into intuitively adjustable parameters, AI tools effectively reduce the creator’s cognitive load. This enables creators to allocate limited cognitive resources toward higher-level aesthetic decision-making and refined artistic expression. Most importantly, the immediate feedback provided by AI establishes an “exploration-feedback-optimization” cycle that closely aligns with the “generate-evaluate” model in creative cognition. Therefore, AI not only assists creation but also serves as an “externalized thinking partner” at the cognitive level, collectively constituting an enhanced form of musical creativity.

### 6.2 Chain mediation effect

Research has found that the impact of AI music tools on musical creativity is not limited to direct effects. Through a chain of mediating mechanisms involving musical self-efficacy and musical emotional intelligence, AI tools can promote creativity at a deeper level. Analysis based on social cognitive theory suggests that AI tools first enhance creators’ musical self-efficacy, thereby promoting creative performance. Specifically, the real-time feedback and professional guidance provided by AI systems enable creators to continuously receive positive confirmation of their abilities. This gradual experience of success effectively reinforces their confidence in their own creative abilities. This increased confidence not only enhances creative motivation but, more importantly, encourages creators to break out of existing creative frameworks and experiment with more innovative musical expressions. Second, enhanced self-efficacy encourages creators to make deeper use of the emotional analysis functions of AI tools, thereby improving their musical emotional intelligence. In this process, AI systems serve as both an auxiliary tool for emotional cognition, helping creators accurately identify emotional characteristics in musical elements, and an extension of emotional expression, enabling creators to achieve more subtle emotional transmission through precise parameter control. This technology-enabled psychological enhancement enables musical works to exhibit richer emotional dimensions and deeper expressive power.

It is worth noting that these two psychological mechanisms do not exist in isolation but form a dynamic, mutually reinforcing system: the use of AI tools enhances self-efficacy, which in turn improves emotional intelligence, ultimately promoting higher levels of musical creativity. In turn, the enhancement of musical creativity reinforces self-efficacy. This finding deepens our understanding of how technology empowers artistic creation. More importantly, it reveals the interactive mechanism between technological and psychological factors in human-machine collaborative creation. From a theoretical perspective, this mechanism explains a new feature of artistic creation in the digital age: technological tools not only expand the boundaries of creative possibilities but also continuously stimulate innovative potential by altering the psychological state of creators. This understanding provides important theoretical basis for the design of future AI music tools and artistic education practices.

### 6.3 Research comparison and contributions

Compared with existing studies, this study breaks through the traditional single perspective of AI tools as “technical intermediaries” in terms of mechanism interpretation. By integrating social cognition theory and the music emotion regulation model (Fitria, 2021; Bandura, 1986; Juslin, 2013), it constructs a dual psychological pathway model of AI tools’ influence on musical creativity. This finding echoes Ularu’s assertion that “digital technology is reshaping the psychological process of artistic creation,” providing a new theoretical framework for understanding the mechanism of human-machine collaborative creation (Ularu, 2020). Second, in terms of variable selection, existing literature mostly focuses on the impact of AI’s technical characteristics on creative efficiency (Chinamanagonda, 2021), while this study reveals two key mediating variables: musical self-efficacy and musical emotional intelligence. This theoretical transfer validates Longuet’s hypothesis that “AI tools drive the evolution of creators’ psychological abilities” and reveals the relationship between technology use and psychological development through empirical data (Longuet-Higgins, 1994). Finally, at the practical guidance level, this study emphasizes that the design of AI music tools should focus on the compatibility between technology and the psychological development of creators. Specifically, developers should incorporate users’ psychological growth patterns into technical design, such as dynamically adjusting tool complexity to match creators’ confidence levels, rather than simply pursuing feature overload. For music education, it is recommended to establish a training system that balances technical application with psychological skills (such as creative confidence and emotional expression), enabling creators to not only master AI tool usage skills but also develop the psychological foundation necessary for long-term innovation. These practical insights provide a clear direction for the “humanization” of AI music technology.

## 7 Conclusion

This study adopts an interdisciplinary approach combining psychology, musicology, and artificial intelligence to explore the innovative application mechanisms of AI technology in music creation and its impact on creativity. The findings reveal that AI, as an intelligent creative assistance tool, not only supports creation through technical means such as lowering technical barriers and providing real-time feedback but, more importantly, promotes the development of creators’ musical creativity through two key psychological pathways: “musical self-efficacy” and “musical emotional intelligence.” This dual mechanism of technological and psychological influence helps creators build stronger creative confidence at the cognitive level and achieves a dynamic balance between technical norms and artistic freedom at the emotional level by making the rules of musical emotional expression explicit.

From a practical perspective, this study has important implications for advancing the United Nations Sustainable Development Goal SDG 4 (Quality Education). The research findings indicate that AI tools, through their unique interactive methods, can effectively enhance learners’ self-efficacy and emotional intelligence—two key psychological abilities that form the core foundation for the development of innovative expression skills. This provides a new technological pathway for achieving inclusive arts education on a global scale: on the one hand, AI-assisted tools can break free from the traditional reliance of music education on teachers and equipment, enabling more learners to access high-quality arts education opportunities; on the other hand, AI-based music education systems designed according to psychological development principles can provide personalized support tailored to the cognitive and emotional characteristics of different learners, truly realizing the educational philosophy of “teaching according to individual aptitude.” Future research could further explore the differentiated effects of AI tools on creative psychology across different cultural contexts, develop more culturally adaptive intelligent education solutions, and contribute to the widespread availability of high-quality music education globally. These findings not only provide theoretical foundations for music educators to integrate AI technology but also guide educational technology developers in designing AI systems that better align with learners’ psychological development needs.

## 8 Shortcomings and outlook

While this study provides initial insights into the psychological mechanisms through which AI music tools influence musical creativity, several limitations should be acknowledged. First, regarding sample characteristics, our data were primarily drawn from Chinese university students, which constrains the cultural generalizability of our findings. The Chinese music education system emphasizes technical mastery and adherence to established repertoire, potentially shaping students’ self-efficacy development through distinct pathways such as greater reliance on instructor validation. In contrast, Western music pedagogy often encourages personal interpretation and improvisation at earlier stages. These cultural differences may lead to varied effects of AI tools on emotional expression and self-efficacy enhancement, warranting further validation of our model’s applicability in Western contexts.

Second, methodological constraints exist. The cross-sectional design cannot adequately capture the dynamic, reciprocal relationship between creators’ psychological capacities and their use of AI tools. Third, measurement limitations should be noted, as the absence of a unified assessment framework for AI technical characteristics hinders precise quantification of how these key variables influence psychological mechanisms.

Future research should advance in several dimensions. First, cross-cultural comparisons should be prioritized by expanding sample diversity to systematically examine potential differences in how self-efficacy and emotional intelligence function within AI-assisted creation across distinct musical education traditions. Second, multidimensional assessment systems should be developed, including standardized measurement tools adapted for AI music creation contexts, potentially incorporating physiological indicators such as EEG and skin conductance with creative behavior logs to establish multimodal evaluation frameworks. Third, longitudinal tracking designs should be implemented to reveal the co-evolutionary pathways of creators’ psychological capacities and artistic styles throughout their creative development. Finally, interdisciplinary integration and practical applications should be promoted by establishing a “music-psychology-computer science” cross-disciplinary research paradigm. This would provide psychological foundations for human-centered AI music system design while facilitating the development of dual-track music education models that integrate “technological empowerment” with “psychological nurturing,” ultimately supporting creators in achieving comprehensive development encompassing technology and art, cognition and emotion in the digital age.

## Data Availability

The raw data supporting the conclusions of this article will be made available by the authors, without undue reservation.

## References

[ref1] Al-Ghawanmeh F. Al-Ghawanmeh M. Obeidat N. Toward an improved automatic melodic accompaniment to Arab vocal improvisation, Mawwāl. In: Proc 9th Conf Interdiscip Musicol-CIM14. (2014):397–400.

[ref2] Alward P. (2023). Musical types and musical flexibility. Acta Anal. 38, 355–369. doi: 10.1007/s12136-022-00518-z

[ref3] Bakht S. Barlow C. (2009). PAPAGEI: an extensible automatic accompaniment system for live instrumental improvisation: ICMC.

[ref4] Bandura A. (1986). Social foundations of thought and action, Englewood Cliffs, NJ: Prentice-Hall. 2:23–28.

[ref5] Bandura A. (2002). Social cognitive theory in cultural context. Appl. Psychol. 51, 269–290. doi: 10.1111/1464-0597.00092

[ref6] Beghetto R. A. (2019). “Structured uncertainty: how creativity thrives under constraints and uncertainty” in Creativity under duress in education? Resistive theories, practices, and actions, ed. C. A. Mullen. Cham, Switzerland: Springer International Publishing, 27–40.

[ref7] Bretan M. Weinberg G. (2016). A survey of robotic musicianship. Commun. ACM 59, 100–109. doi: 10.1145/2818994

[ref8] Burnard P. (2012). “Musical creativity” in Oxford handbook music Educ, New York, NY: Oxford University Press, 2, 319.

[ref9] Chen L. (2025). Unlocking the beat: how AI tools drive music students' motivation, engagement, creativity and learning success. Eur. J. Educ. 60:e12823. doi: 10.1111/ejed.12823

[ref10] Chinamanagonda S. (2021). AI-driven performance testing AI tools enhancing the accuracy and efficiency of performance testing. Adv. Comput. Sci. 4:1–20.

[ref11] Deliège I. Wiggins G. A. (2006). Musical creativity: multidisciplinary research in theory and practice. Hove, UK: Psychol Press.

[ref12] Deruty E. Grachten M. Lattner S. Nistal J. Aouameur C. (2022). On the development and practice of AI technology for contemporary popular music production. Trans. Int. Soc. Music Inf. Retr. 5:35. doi: 10.5334/tismir.100

[ref13] Doppelt Y. (2009). Assessing creative thinking in design-based learning. Int. J. Technol. Des. Educ. 19, 55–65. doi: 10.1007/s10798-006-9008-y

[ref14] Fitria T. N. (2021). “Artificial intelligence (AI) in education: using AI tools for teaching and learning process” in Prosiding Seminar Nasional & Call for Paper STIE AAS” are unavailable in standard academic databases. Surakarta, Indonesia, 134–147.

[ref15] Fornell C. Larcker D. F. (1981). Evaluating structural equation models with unobservable variables and measurement error. J. Mark. Res. 18, 39–50. doi: 10.1177/002224378101800104

[ref16] Gardner H. E. (2008). Multiple intelligences: new horizons in theory and practice. New York, NY: Basic Books.

[ref17] Gioti A. M. (2020). From artificial to extended intelligence in music composition. Organised Sound 25, 25–32. doi: 10.1017/S1355771819000438

[ref18] Gleason M. E. Musical aptitude and emotional intelligence. Doctoral dissertation. (2014)

[ref19] Gundlach R. H. (1935). Factors determining the characterization of musical phrases. Am. J. Psychol. 47, 624–643. doi: 10.2307/1416007

[ref20] Hewitt M. P. (2015). Self-efficacy, self-evaluation, and music performance of secondary-level band students. J. Res. Music. Educ. 63, 298–313. doi: 10.1177/0022429415595611

[ref21] Huang Y. S. Yang Y. H.. Pop music transformer: beat-based modeling and generation of expressive pop piano compositions. In: Proc 28th ACM Int Conf Multimed. (2020):1180–1188.

[ref22] Jia C. Ayob A. (2025). The benefits of emotional intelligence in developing a strong foundation for musical experience. ICCCM J. Soc. Sci. Hum. 4, 21–25. doi: 10.53797/icccmjssh.v4i1.4.2025

[ref23] Jiang J. (2024). Impact of music learning on students' psychological development with mediating role of self-efficacy and self-esteem. PLoS One 19:e0309601. doi: 10.1371/journal.pone.0309601, PMID: 39226287 PMC11371213

[ref24] Jiang H. Cheong K. W. Tan W. H. (2024). Development and validation of a measure assessing children's creative practice ability in music. Think. Skills Creat. 51:101446. doi: 10.1016/j.tsc.2023.101446, PMID: 41130138

[ref25] Juslin P. N. (2013). From everyday emotions to aesthetic emotions: towards a unified theory of musical emotions. Phys Life Rev 10, 235–266. doi: 10.1016/j.plrev.2013.05.008, PMID: 23769678

[ref26] Kaufman J. C. (2012). Counting the muses: development of the Kaufman domains of creativity scale (K-DOCS). Psychol. Aesthet. Creat. Arts 6:298.

[ref27] Law K. S. Wong C. S. Song L. J. (2004). The construct and criterion validity of emotional intelligence and its potential utility for management studies. J. Appl. Psychol. 89, 483–496. doi: 10.1037/0021-9010.89.3.483, PMID: 15161407

[ref28] Lemons G. (2010). Bar drinks, rugas, and gay pride parades: is creative behavior a function of creative self-efficacy? Creat. Res. J. 22, 151–161. doi: 10.1080/10400419.2010.481502

[ref29] Liu C. H. Ting C. K. (2016). Computational intelligence in music composition: a survey. IEEE Trans. Emerg. Top. Comput. Intell. 1, 2–15. doi: 10.1109/TETCI.2016.2642200

[ref30] Livingstone S. R. Muhlberger R. Brown A. R. Thompson W. F. (2010). Changing musical emotion: a computational rule system for modifying score and performance. Comput. Music. J. 34, 41–64. doi: 10.1162/comj.2010.34.1.41

[ref31] Longuet-Higgins H. C. (1994). Artificial intelligence and musical cognition. Philos. Trans. R. Soc. Lond. A. Phys. Eng. Sci. 349, 103–113. doi: 10.1098/rsta.1994.0116, PMID: 40742943

[ref32] López-González M. Limb C. J. (2012). Musical creativity and the brain. Cerebrum Dana Forum Brain Sci. 2012, 2. PMC3574774 23447788

[ref33] Luszczynska A. Schwarzer R. (2015). Social cognitive theory. Fac Health Sci Publ, 2015:225–251.

[ref34] McPherson G. E. McCormick J. (2006). Self-efficacy and music performance. Psychol. Music 34, 322–336.

[ref35] Merrick B. M. The relationship between self-efficacy and self-regulated behaviour within a secondary school music technology based creative learning environment. Doctoral dissertation, UNSW Sydney (2006)

[ref36] Monelle R. (1997). Musical uniqueness as a function of the text. Appl. Semiot. 2:56.

[ref37] Motukeeva A. Azhibaeva A. Kulbachaev D. Abdyrakunova Z. Toloev M. (2024). Formation of creative thinking among students in the digital educational environment. E-Learn Digit Media.:20427530241307671. doi: 10.1177/20427530241307671

[ref38] Nehra E. Artificial intelligence in modern times. In: Int Conf Recent Innov Sci Eng Manag (2015)

[ref39] Nemt-Allah M. Khalifa W. Badawy M. Elbably Y. Ibrahim A. (2024). Validating the ChatGPT usage scale: psychometric properties and factor structures among postgraduate students. BMC Psychol. 12:497. doi: 10.1186/s40359-024-01983-4, PMID: 39317930 PMC11423513

[ref40] Neubauer A. C. Freudenthaler H. H. (2005). “Models of emotional intelligence” in Emot Intell Int Handb, eds. R. Schulze, and R. D. Roberts. Cambridge, MA: Hogrefe & Huber, 31–50.

[ref41] Otchere E. D. (2014). Music and emotion: a study of the relationship between musical preference and emotional intelligence. Doctoral dissertation. University of Cape Coast.

[ref42] Pachet F. (2006). “Creativity studies and musical interaction” in Musical creativity, eds. I. Deliège, and G. A. Wiggins, Hove, UK: Psychology Press, 363–374.

[ref43] Qureshi O. (2023). Artistic innovation and creativity: driving forces of human progress. J. Relig. Soc. 1, 1–16.

[ref44] Resnicow J. E. Salovey P. Repp B. H. (2004). Is recognition of emotion in music performance an aspect of emotional intelligence? Music. Percept. 22, 145–158. doi: 10.1525/mp.2004.22.1.145

[ref45] Rezwana J. Maher M. L. (2023). Designing creative AI partners with COFI: a framework for modeling interaction in human-AI co-creative systems. ACM Trans. Comput. Hum. Interact. 30, 1–28. doi: 10.1145/3519026

[ref46] Ritchie L. Williamon A. (2011). Measuring distinct types of musical self-efficacy. Psychol. Music 39, 328–344. doi: 10.1177/0305735610374895

[ref47] Ritchie L. Williamon A. (2012). Self-efficacy as a predictor of musical performance quality. Psychol. Aesthet. Creat. Arts 6, 334–340. doi: 10.1037/a0029619

[ref48] Roberts A. Engel J. Raffel C. Hawthorne C. Eck D. (2018). A hierarchical latent vector model for learning long-term structure in music. Int Conf Mach learn, 4364–4373.

[ref49] Runco M. A. Jaeger G. J. (2012). The standard definition of creativity. Creat. Res. J. 24, 92–96. doi: 10.1080/10400419.2012.650092

[ref50] Ryan T. G. Brown K. (2012). Musical creativity: measures and learning. J. Elem. Educ. 22, 105–120.

[ref51] Schwarzer R. Luszczynska A. (2008). Self efficacy. Handb. Posit. Psychol. Assess. 2, 7–217.

[ref52] Shabani K. Khatib M. Ebadi S. (2010). Vygotsky's zone of proximal development: instructional implications and teachers' professional development. Engl. Lang. Teach. 3, 237–248. doi: 10.5539/elt.v3n4p237

[ref53] Shih Y. J. Wu S. L. Zalkow F. Müller M. Yang Y. H. (2022). Theme transformer: symbolic music generation with theme-conditioned transformer. IEEE Trans. Multimed. 25, 3495–3508. doi: 10.1109/TMM.2022.3161851

[ref54] Su W. S. Hsu C. C. Huang C. H. Chang L. F. (2018). Setting attributes and revisit intention as mediated by place attachment. social behavior and personality: an international journal, 46(12), 1967–1981.Guilford JP. Creativity: yesterday, today and tomorrow. J. Creat. Behav. 1967;1:3–14.

[ref55] Turing A. M. (1980). Computing machinery and intelligence. Creat. Comput. 6, 44–53.

[ref56] Ularu N. (2020). “The impact of technology in reshaping artistic education” in Art Res Contemp Chall, eds. M. R. Oginska, and J. Zwolinska. Warsaw, Poland: Sciendo, 16–27.

[ref57] Vadlamudi E. Curha N. (2019). A study on emotional intelligence and creativity among musicians. Journal of Arts, Culture, Philosophy, Religion, Language and Literature e-ISSN: 2457-0346, 3, 30-38. Available at online: http://www.gcmishraedu.com/Publications.html

[ref58] Vuoskoski J. K. Thompson W. F. McIlwain D. Eerola T. (2011). Who enjoys listening to sad music and why? Music. Percept. 29, 311–317. doi: 10.1525/mp.2012.29.3.311

[ref59] Waddington J. (2023). Self-efficacy. ELT J. 77, 237–240. doi: 10.1093/elt/ccac046

[ref60] Wang X. Li P. (2024). Assessment of the relationship between music students' self-efficacy, academic performance and their artificial intelligence readiness. Eur. J. Educ. 59:e12761. doi: 10.1111/ejed.12761

[ref61] Webster P. R. (2002). Creativity and music education: creative thinking in music: advancing a model. Creat Music Educ 1:16.

[ref62] Wu S. L. Yang Y. H. (2023). Musemorphose: full-song and fine-grained piano music style transfer with one transformer VAE. IEEE/ACM Trans. Audio Speech Lang. Process. 31, 1953–1967. doi: 10.1109/TASLP.2023.3270726

[ref63] Zarza-Alzugaray F. J. Casanova O. McPherson G. E. Orejudo S. (2020). Music self-efficacy for performance: an explanatory model based on social support. Front. Psychol. 11:1249. doi: 10.3389/fpsyg.2020.01249, PMID: 32670146 PMC7330084

[ref64] Zhang L. (2025). Compositional tools based on artificial intelligence for choral artistic education: enhancing creative skills in choral arrangements. Think. Skills Creat. 56:101768. doi: 10.1016/j.tsc.2025.101768

[ref65] Zimmerman B. J. (2000). “Attaining self-regulation: a social cognitive perspective” in Handbook of self-regulation, eds. M. Boekaerts, P. R. Pintrich, and M. Zeidner. San Diego, CA: Academic Press, 13–39.

[ref66] Zulić H. (2019). How AI can change/improve/influence music composition, performance and education: three case studies. INSAM J Contemp Music Art Technol. 2, 100–114.

